# Research progress on the correlation between biological rhythms and the blood-brain barrier after ischemic stroke

**DOI:** 10.3389/fneur.2025.1627172

**Published:** 2025-09-26

**Authors:** Yuanchen Liao, Lei Luo, Qiang Ma, Xiaofeng Gao, Yao Chen, Siyang Yan, Menghao He, Lijuan Liu, Desheng Zhou

**Affiliations:** ^1^Hunan University of Chinese Medicine, Changsha, Hunan, China; ^2^The Key Laboratory of Hunan Province for Integrated Traditional Chinese and Western Medicine on Prevention and Treatment of Cardio-Cerebral Diseases, College of Integrated Traditional Chinese and Western Medicine, Hunan University of Chinese Medicine, Changsha, Hunan, China; ^3^The First Affiliated Hospital of Hunan University of Chinese Medicine, Changsha, Hunan, China

**Keywords:** biological rhythms, ischemic stroke, blood-brain barrier, angiogenesis, immune system, neuroendocrine system

## Abstract

Biological rhythms play a critical role in regulating human physiology and have been implicated in the onset, progression, and recovery of ischemic stroke (IS). This review summarizes recent experimental and clinical studies that associate circadian regulation with post-stroke blood–brain barrier (BBB) repair, focusing on the role of molecular clock components. Core clock components, including BMAL1 and CLOCK, influence BBB integrity by regulating tight junction protein expression, angiogenesis, neuroimmune responses, and neuroendocrine signaling. Finally, we discuss emerging chronotherapeutic strategies that integrate circadian biology into stroke rehabilitation.

## 1 Introduction

Ischemic stroke (IS), also referred to as cerebral infarction, arises from reduced or interrupted cerebral blood flow, leading to ischemic-hypoxic necrosis of brain tissue and subsequent neurological deficits. According to the Global Burden of Disease 2021 study, IS ranks as the second leading cause of death worldwide and remains a major cause of long-term disability (1). The pathophysiology of IS is initiated by cerebral hypoperfusion and progresses through multiple interconnected processes, including excitotoxicity (2–4), oxidative stress (5, 6), and neuroinflammation (7, 8). Acute cerebral ischemia/hypoxia rapidly depletes ATP, which in turn provokes persistent neuronal hyperexcitation and widespread apoptosis. At the same time, excessive generation of reactive oxygen species (ROS) promotes apoptotic signaling and cellular dysfunction, while activation of innate immunity maintains cytokine and chemokine release, thereby amplifying ischemic injury (9). Current therapeutic strategies for IS aim to restore cerebral perfusion as quickly as possible, mainly via intravenous thrombolysis with recombinant tissue plasminogen activator (rt-PA) or by endovascular mechanical thrombectomy. When applied within the therapeutic window, these interventions improve blood and oxygen delivery to the ischemic penumbra, help preserve neuronal function, and reduce long-term disability (10, 11). However, because of the narrow therapeutic window and the risk of hemorrhagic complications, fewer than 10% of patients receive rt-PA, and less than half achieve successful reperfusion (12, 13). This underscores the urgent requirement for safer and more effective treatment options.

Disruption of the blood–brain barrier (BBB) is a key pathological feature of IS, primarily caused by degradation of tight junction (TJ) proteins and increased transcytosis, leading to vascular hyperpermeability (14). BBB breakdown triggers the release of damage-associated molecular patterns (DAMPs), such as vascular endothelial growth factor (VEGF), matrix metalloproteinases (MMPs), and heat shock proteins (HSPs), from ischemic tissue, and promotes immune cell infiltration that accelerates neuronal necrosis (15). Necrotic neurons further release DAMPs, enhancing chemokine secretion by immune cells and perpetuating a cycle of vascular injury, neuroinflammation, and neuronal death. This pathological cascade aggravates ischemia–reperfusion injury and promotes secondary complications, including vasogenic edema and hemorrhagic transformation (16–18). Clinical studies have linked severe BBB disruption to unfavorable IS outcomes, such as higher NIH Stroke Scale (NIHSS) scores, poorer functional recovery on the modified Rankin Scale (mRS), and increased mortality (19). Therefore, therapies aimed at preserving BBB integrity may help reduce neurological deficits and improve long-term outcomes in patients with IS.

The BBB is a specialized neurovascular interface that regulates molecular and ionic exchange between the systemic circulation and the central nervous system (CNS), thereby maintaining cerebral homeostasis (20). The neurovascular unit constituting the BBB primarily comprises brain microvascular endothelial cells (BMECs), astrocytes, and pericytes interacting with neurons and microglia. These cellular components interact structurally and molecularly to establish and regulate barrier function (21, 22). Selective permeability of the BBB depends on tight junction complexes between endothelial cells, which are composed of transmembrane proteins (claudins, occludin, and junctional adhesion molecules [JAMs]) and cytoplasmic scaffolding proteins of the zona occludens (ZO) family. Together, these structures restrict paracellular diffusion. In addition, ATP-dependent transporters such as P-glycoprotein, together with vesicular mechanisms including adsorptive and receptor-mediated transcytosis, control transcellular molecular transport (23–25). Key mechanisms of BBB transport include receptor-mediated transcytosis (e.g., via transferrin receptors), efflux mediated by ATP-binding cassette (ABC) transporters such as BCRP, and increased paracellular permeability when tight junctions are disrupted (26). Mesenchymal stem cell-derived extracellular vesicles have been shown to help preserve BBB integrity and reduce the risk of hemorrhagic transformation (27). This is clinically important since symptomatic intracranial hemorrhage (sICH), although reported in only 2–7% of thrombolysis cases, is responsible for the majority of thrombolysis-related deaths (28). Recent studies have shown that endothelial clock genes (CLOCK/BMAL1) regulate the expression of tight junction proteins such as CLDN5 and OCLN, suggesting that circadian rhythms contribute to endogenous protection of BBB function (29).

Circadian rhythms are endogenous 24-h cycles regulated by the suprachiasmatic nucleus (SCN) in the hypothalamus, which synchronizes peripheral oscillators across mammalian tissues (30). Cell-autonomous rhythms are generated by transcription–translation feedback loops (TTFLs) involving core clock genes such as Clock, Bmal1, Per, and Cry (31). Clock genes are broadly expressed, not only in the SCN but also in other brain regions and peripheral organs (32, 33). At the molecular level, BMAL1 and CLOCK form heterodimers (CLOCK::BMAL1) that regulate transcription of downstream genes by binding to E-boxes, D-boxes, and Rev-erb/ROR response elements (34). Disruption of circadian rhythms contributes to pathological processes such as impaired DNA damage repair (35), altered metabolic and oxidative stress regulation (36), and dysregulated inflammatory and immune responses (37). Moreover, Circadian genes also regulate angiogenesis by influencing endothelial and pericyte functions, through mechanisms such as modulation of angiogenic factors (38), basement membrane degradation (39), extracellular matrix remodeling (40), and regulation of endothelial migration, proliferation, and pericyte recruitment (41).

Circadian rhythms influence the onset, progression, and clinical outcomes of IS by regulating diurnal blood pressure patterns, vascular tone, and platelet activity (42). Ambulatory blood pressure monitoring (ABPM) has provided strong evidence linking circadian disruption to stroke incidence. Abnormal circadian blood pressure rhythms are recognized as an independent risk factor for IS (43, 44). In rodent models, six weeks of environmental circadian disruption (ECD) increased infarct size and enhanced neuroinflammatory responses, thereby worsening stroke severity (45). Circadian-regulated molecules such as TNF-α, leptin, β-amyloid, delta sleep-inducing peptide (DSIP), and prostaglandin D_2_ (PGD_2_) are expressed in the central nervous system and may influence stroke pathogenesis by altering BBB circadian dynamics (46–50). However, the mechanisms by which circadian rhythms regulate BBB repair after ischemia and influence neurological recovery are still unclear. Further investigation in this area could provide the basis for chronotherapeutic approaches in stroke and identify novel strategies to protect the BBB through circadian regulation.

## 2 Circadian rhythms directly regulate BBB damage and repair following IS

### 2.1 Circadian rhythms directly regulate endothelial cell function

Endothelial cells (ECs) of the BBB display circadian rhythmicity and contain an intrinsic circadian regulatory system (51). At the molecular level, circadian regulation in ECs is governed by a transcription–translation feedback loop involving core clock genes such as CLOCK and BMAL1. Pan et al. reported that leptin transport across the BBB varies with time of day, being significantly higher at night than during the day (46). This variation correlates with circadian changes in the expression of endothelial transporters. Similarly, expression of the glucose transporter GLUT1 peaks during the circadian active phase (52). Members of the ABC efflux transporter family, which are highly expressed in BBB ECs, are regulated by both endothelial circadian clocks and neuronal activity patterns (53). Recent approaches integrating chemogenetic modulation (e.g., clozapine-N-oxide induction) with fluorescence-activated cell sorting and transcriptomic profiling have enabled detailed mapping of activity-dependent gene networks in endothelial cells, key BBB transporters such as P-glycoprotein show rhythmic expression coordinated with core clock genes, generating time windows that favor CNS drug penetration (54, 55). Transporter activity peaks during the circadian active phase (daytime in humans) and decreases during rest phases, inversely related to neuronal activity. Clinical studies have reported better safety outcomes for intravenous thrombolysis administered between noon and midnight compared with treatments given in the early morning (06:00–18:00) (56). These findings suggest that circadian timing should be considered in optimizing stroke treatment strategies.

ECs repair after ischemic stroke involves migration, proliferation, tube formation, and restoration of barrier function, each subject to time-dependent regulation that influences the course of BBB recovery (57). Astone et al. showed that the core clock gene BMAL1 regulates EC proliferation by modulating cell-cycle regulators, particularly cyclin D1 (58). In BMAL1-knockout mice, EC repair is impaired, leading to delayed BBB restoration after stroke (59). These findings indicate that clock genes are essential for endothelial regeneration. Pulido et al. observed that cerebrovascular EC proliferation depends on circadian phase and aligns with oscillations in core clock gene expression (55). When BMAL1 levels are high, ECs demonstrate increased migration and tube formation, which accelerates BBB repair. Recent studies suggest that cryptochrome (CRY) proteins regulate EC repair by coordinating the timing of proliferation, migration, and tube formation, thereby supporting neovascularization and vascular maturation (60). This regulation helps limit BBB hyperpermeability, providing a mechanism by which circadian disruption impairs endothelial repair and disturbs BBB homeostasis. Coordinated circadian control of EC repair is required to maintain barrier integrity, whereas its disruption results in persistent vascular leakage and impaired neurovascular recovery after stroke.

### 2.2 Circadian rhythms directly regulate tight junction proteins

Circadian rhythms influence the expression of genes and proteins associated with tight junctions at both the transcriptional and translational levels. Among these, Claudin-5, Occludin, and zonula occludens-1 (ZO-1) are central to maintaining the structural integrity and function of tight junctions (23, 61).Claudin-5, a principal protein in inter-endothelial tight junctions, determines BBB selective permeabilitys (62). Occludin has been proposed as a marker of tight junction integrity, with reduced levels correlating with BBB disruption and cerebral edema (63). ZO-1 links transmembrane tight junction proteins to the actin cytoskeleton, and its expression and phosphorylation status are important for junction assembly and maintenance (64–66).Recent studies show that the expression of these tight junction proteins follows circadian oscillations (67).

Spadoni et al. reported that expression of claudin-5 and occludin depends on the core clock gene BMAL1. In wild-type mice, occludin mRNA shows clear circadian oscillation, which is lost in rhythm-disrupted mice and accompanied by impaired barrier integrity (68). Kyoko et al. found that claudin-5 expression in the intestinal vasculature is higher at night (active phase) than during the day (rest phase) (29). These findings support circadian regulation of claudin-5 (69) and suggest that the CLOCK/BMAL1 heterodimer regulates its transcription through E-box elements. Jensen et al. showed that loss of BMAL1 reduces claudin-5 expression and increases BBB permeability (70). Expression of the clock repressor Period2 (Per2) is inversely correlated with claudin-5 levels, supporting circadian regulation of tight junction dynamics (71). After ischemic stroke, tight junction repair is time-dependent, with efficiency differing across circadian phases (69). In rhythm-synchronized mice, occludin and claudin-5 show periodic changes in expression during the first 72 h after injury, whereas such oscillations are absent in rhythm-disrupted mice (72, 73). Together, these findings suggest that circadian regulation of tight junction proteins is an important mechanism for maintaining BBB integrity, and that targeting core clock components may help promote BBB recovery after ischemic stroke.

## 3 Circadian rhythms promote post-IS BBB repair through angiogenesis regulation

### 3.1 Circadian rhythms modulate pericyte phenotypic reprogramming to restore BBB integrity after IS

Angiogenesis after stroke is an important stage of BBB restoration, involving endothelial cell proliferation, migration, and pericyte recruitment. Circadian regulation of angiogenesis has been linked to core clock genes such as CLOCK and BMAL1 (70, 74). Post-stroke angiogenesis shows spatiotemporal heterogeneity (75). Immature neovessels often remain hyperpermeable, which aggravates BBB dysfunction, vasogenic edema, and neuroinflammation. Pericytes contribute to CNS homeostasis by regulating microcirculatory flow, controlling leukocyte entry, clearing neurotoxic metabolites, modulating circadian endothelial gene expression, and maintaining astrocytic end-foot polarization (76). As specialized capillary mural cells, they stabilize newly formed vessels during both developmental angiogenesis and post-injury repair. Functional vascular maturation requires adequate pericyte recruitment and continuous vessel wall coverage. Loss of pericytes alters endothelial gene expression and promotes pathological angiogenesis with persistent hyperpermeability (77). Jidigam et al. showed that BMAL1 regulates pericyte recruitment (78). In BMAL1-knockout mice, pericytes progressively detach, extracellular matrix deposition is reduced, and tight junctions disassemble, leading to unstable neovasculature and loss of BBB integrity (79). BMAL1 also regulates vascular maturation and stabilization by controlling the transcription of adhesion molecules and cytoskeletal regulators (80).

### 3.2 Circadian rhythms regulate VEGF–Mediated BBB repair following IS

VEGF shows circadian-regulated expression mediated by the molecular clock (81). It influences endothelial cell behavior and pericyte recruitment, and is a major determinant of angiogenic responses after stroke. Excessive VEGF expression enhances neuroinflammation and promotes the formation of immature, hyperpermeable vessels, resulting in tissue injury and worsening BBB dysfunction (82).BMAL1 binds E-box elements in the VEGFA promoter to activate its transcription, which stimulates endothelial proliferation, migration, and differentiation (83). Through VEGF signaling, BMAL1 helps regulate the balance between tip and stalk cells, which is required for stable vascular network formation. Conversely, the clock repressors PER2 and CRY1 suppress hypoxia-induced VEGFA transcription, contributing to circadian oscillations in VEGF expression (84). In hindlimb ischemia models, both genetic and environmental circadian disruption impair reparative neovascularization (74). Clinical studies report that shift workers show delayed vascular recovery after stroke, associated with disrupted VEGF rhythmicity and clock gene dysregulation (85).These findings suggest that the molecular clock regulates endothelial function and vascular repair by modulating VEGF signaling over time. Koyanagi et al. showed that BMAL1 coordinates diurnal VEGFA expression via hypoxia-inducible factor 1α (HIF-1α) in tumor models, and this mechanism also contributes to vascular repair after stroke (83). Fibroblast growth factors (FGFs), such as FGF21, show circadian expression and support vascular repair by enhancing VEGF signaling and promoting the activity of endothelial progenitor cells (EPCs) (86, 87).Together, these data suggest that therapeutic strategies targeting both clock components (CLOCK/BMAL1 or PER/CRY) and angiogenic factors (VEGFA/FGF) could enhance BBB recovery and improve outcomes after stroke. Such chronotherapeutic approaches may help preserve physiological angiogenic rhythms while limiting pathological vascular permeability in ischemic stroke.

## 4 Circadian rhythms regulate post-IS BBB repair via immune system modulation

### 4.1 Circadian rhythms orchestrate immune cell-mediated BBB repair following IS

After stroke, BBB dysfunction involves several inflammatory mechanisms, including increased matrix metalloproteinases (MMP-2/9), excessive ROS production, polarization of microglia toward pro-inflammatory states, and infiltration of peripheral leukocytes (18, 88–91). Thus, neuroinflammation strongly influences BBB recovery, and this process is under circadian regulation. The molecular clock regulates immune function by controlling leukocyte proliferation and migration, modulating phagocytic and cytotoxic activity, and influencing immune cell activation states. As a result, circadian regulation affects key immune processes such as pathogen defense, tissue surveillance, and homeostasis (92). Disruption of circadian rhythms disturbs the balance between pro- and anti-inflammatory cytokines, partly through altered immune cell trafficking and dysregulated clock proteins such as REV-ERBα/NR1D1 (93).Innate immune cells such as macrophages and NK cells contain functional circadian clocks, with the CLOCK:BMAL1 heterodimer regulating phagocytosis, cytokine production (e.g., IL-1β, TNF-α), and antimicrobial activity (94). Endothelial-specific BMAL1 deletion disrupts the rhythmic trafficking of immune cells to lymph nodes, highlighting the importance of circadian rhythms in regulating immune cell positioning (95).

The circadian system contributes to BBB repair after ischemic stroke by regulating neuroimmune interactions in a time-dependent manner across different pathological phases. During the acute phase (< 72 h post-stroke), microglia show circadian-dependent activation patterns, influencing the release of pro-inflammatory mediators (IL-6, ROS), modulating MMP-9 secretion, and thereby affecting the extent of BBB disruption (96, 97). Yenari et al. showed that suppression of microglial activation with minocycline reduced TNF-α and IL-1β secretion, promoted endothelial repair, decreased infarct volume and BBB leakage, and improved neurological outcomes (98). BMAL1 regulates the temporal pattern of MMP-9 expression by controlling neutrophil infiltration rhythms (99). Circadian-driven migration of Treg cells helps suppress excessive inflammation while maintaining MMP-9 activity, which supports BBB integrity (100, 101). Neurovascular unit crosstalk is also influenced by circadian regulation. Monocytes and macrophages promote endothelial proliferation and tight junction reassembly during specific circadian phases (102), while astrocyte-derived rhythmic inflammatory factors, such as basic fibroblast growth factor, can worsen BBB dysfunction through peripheral–central signaling (103). Novel immunotherapies that penetrate the BBB, such as neutrophil-hitchhiked bacterial outer membrane vesicles, are being investigated to enhance brain delivery of neuroprotective agents in stroke (104). Unresolved issues include how clock genes influence immune cell activation thresholds (e.g., Treg suppression) and what circadian windows (e.g., ZT4–8) are optimal for immunomodulation. Clarifying these mechanisms may guide the development of chronotherapy strategies for BBB repair.

### 4.2 Circadian rhythms regulate inflammatory factor-mediated BBB repair following IS

Ischemia and hypoxia trigger a neuroinflammatory cascade in which the release of pro-inflammatory mediators disrupts the BBB. The resulting increase in BBB permeability further amplifies inflammation and aggravates neuronal injury. For example, TNF-α downregulates tight junction proteins such as occludin, thereby increasing endothelial permeability (105). IL-1β induces MMP-2/9-mediated degradation of basement membrane collagen (106). IL-6 upregulates VEGF expression and promotes vascular leakage (107), while IL-8 enhances neutrophil infiltration and aggravates BBB dysfunction (108). The secretion of pro-inflammatory cytokines follows circadian rhythms (109). Pharmacological and genetic studies in macrophages show that REV-ERBα links the circadian clock to inflammatory signaling by modulating the expression of innate immune genes such as IL-6 (110, 111). Candelario-Jalil et al. reported that anti-TNF-α therapy was more effective when administered during the nocturnal phase, suggesting that circadian regulation influences TNF-α-mediated inflammation (112). Ding et al. showed that BMAL1 regulates transcriptional activity by binding to cis-regulatory elements within inflammatory gene promoters, thereby controlling their circadian expression (113). Loss of BMAL1 disrupts circadian cytokine oscillations and prolongs inflammatory responses. Although the molecular mechanisms linking circadian rhythms to inflammatory mediators are not fully understood, circadian disruption is known to disturb cytokine regulation. These findings suggest that chrono-targeted strategies, such as timed inhibition of inflammatory pathways and preservation of endothelial tight junctions, may help reduce secondary injury after ischemic stroke.

## 5 Circadian rhythms regulate post-IS BBB repair via the neuroendocrine system

### 5.1 The neuroendocrine-circadian axis

The neuroendocrine system acts as a key interface linking circadian regulation to BBB homeostasis and contributes to barrier repair after ischemic stroke. As the main integrator of neural and hormonal signaling, it exhibits circadian oscillations in both hormone secretion and regulatory activity. The hypothalamus functions as the central regulatory hub, where specialized neurosecretory cells translate synaptic inputs into timed hormonal releases, particularly glucocorticoids under circadian control. Under the control of the SCN, these hypothalamic cells synchronize peripheral tissue clocks and help maintain systemic circadian coherence (114, 115). Recent evidence suggests that this temporal regulation involves not only the hypothalamic–pituitary–adrenal axis but also mesolimbic reward pathways, where circadian fluctuations in monoaminergic neurotransmission (particularly dopamine and serotonin) influence endocrine responses to environmental cues (116, 117). Experimental studies show bidirectional interactions between circadian and neuroendocrine systems, where neurotransmitter imbalances such as dopamine dysregulation impair clock function, and disruption of clock genes leads to neuroendocrine deficits (118, 119). Together, molecular clocks, neural circuits, and hormonal signals form an integrated neuroendocrine–circadian axis that coordinates physiological homeostasis and modulates stress responses.

### 5.2 The hypothalamic–pituitary–adrenal (HPA) axis

The neuroendocrine system regulates BBB integrity through signaling pathways that show clear circadian control (120). The HPA axis, functioning as the neuroendocrine system's master regulator, exhibits intrinsic circadian oscillations in both its basal tone and stress-responsive activity (121).Key HPA effector molecules, particularly glucocorticoids and angiotensin II, modulate BBB permeability in a phase-dependent manner by regulating tight junction proteins (claudin-5, ZO-1) and vascular permeability factors such as VEGF (122–125). Endogenous glucocorticoid secretion follows a conserved circadian pattern across species, with diurnal organisms (e.g., humans) showing peak concentrations during active phases (morning) and nocturnal species (e.g., rodents) displaying maximal levels during their active period (evening) (126).In humans, peak circadian cortisol secretion (approximately 06:00–10:00h) (127) temporally coincides with maximal expression of tight junction proteins (occludin, claudin-5), and optimal BBB integrity. Conversely, the nadir phase (approximately 22:00–02:00 h) is characterized by decreased junctional protein expression and elevated paracellular permeability (76, 128).This circadian variation represents an adaptive mechanism aligning BBB function with metabolic demands. The HPA axis also regulates circadian neuroimmune interactions. Glucocorticoids perform dual chrono-regulatory functions: entraining circadian oscillations in cytokine production (129–132), and exerting phase-dependent immunosuppression through inhibition of pro-inflammatory transcription factors (including NF-κB) (133–136).Preserved circadian cortisol rhythmicity following stroke acts as an endogenous brake on neuroinflammation, thereby limiting persistent BBB dysfunction (137).Clinical investigations demonstrate that blunted circadian cortisol rhythmicity (non-dipping pattern) shows significant positive correlation with both the extent and progression of post-stroke BBB pathology (138).These findings suggest that targeting clock genes to optimize the timing of neuroendocrine factor release (e.g., aligning glucocorticoid peaks with ischemic injury cycles) may provide a strategy for BBB stabilization.

### 5.3 Circadian rhythms modulate melatonin-mediated BBB repair after IS

Melatonin is an indoleamine hormone secreted by the pineal gland in a circadian pattern (139, 140). Melatonin has been shown to exert neuroprotective effects through anti-inflammatory and antioxidant actions, reduction of cerebral edema, preservation of cognitive function, and activation of endogenous repair mechanisms (141–145). Wang et al. reported that melatonin treatment reduced brain water content and improved BBB integrity compared with untreated controls (146). Experimental studies show that nighttime administration of melatonin increases the expression of occludin, claudin-5, and ZO-1, stabilizes the cytoskeleton, and reduces BBB leakage after ischemia. The protective effect is strongest when treatment is synchronized with endogenous secretion rhythms (26). Melatonin also improves endothelial function and protects against excitotoxicity-induced BBB disruption in neonatal rats (147). At the molecular level, melatonin directly binds to MMP-9, thereby inhibiting its activity and reducing ischemia/reperfusion-induced BBB hyperpermeability (148, 149). Together, these findings suggest that melatonin may serve as a therapeutic agent to mitigate post-ischemic BBB injury. Clinically, melatonin has been reported to reduce the risk of hemorrhagic transformation after tissue plasminogen activator (t-PA) therapy in ischemic stroke (150, 151), suggesting a potential role in improving thrombolytic safety through modulation of BBB permeability.

### 5.4 Circadian regulation of autonomic nervous system (ANS) activity to attenuating BBB dysfunction of IS

Sympathetic and parasympathetic activities exhibit circadian rhythmicity and modulate cerebrovascular function through neurovascular coupling mechanisms (152, 153).Sympathetic activity predominates during the daytime, whereas parasympathetic activity is enhanced at night (154). The vagus nerve, as the main parasympathetic effector, shows marked circadian fluctuations in activity (155). Clinical studies indicate that circadian disruption, such as shift work, attenuates the anti-inflammatory effects of vagal activity (156). Preclinical evidence demonstrates that non-invasive vagus nerve stimulation (nVNS) exerts neuroprotective effects, preserving BBB integrity and reducing infarct volume after ischemic injury (157). When applied during peak activity periods (nocturnal phase), VNS suppresses pro-inflammatory gene expression (e.g., IL-6), reduces inflammatory responses, and enhances BBB integrity through upregulation of ZO-1 and occluding (152, 158). Clinical data further reveal an inverse correlation between heart rate variability (HRV, an indicator of vagal activity) and the severity of BBB damage, suggesting that vagal activity contributes to BBB regulation through cerebrovascular dilation and hemodynamic control (159).

The molecular and cellular mechanisms by which circadian rhythms regulate neuroendocrine signaling are not yet fully understood, representing a priority for future investigation. Advancing chronotherapeutic delivery systems for neuroendocrine modulators may enable temporally optimized treatment strategies for ischemic stroke.

## 6 Summary

**Figure 1:**
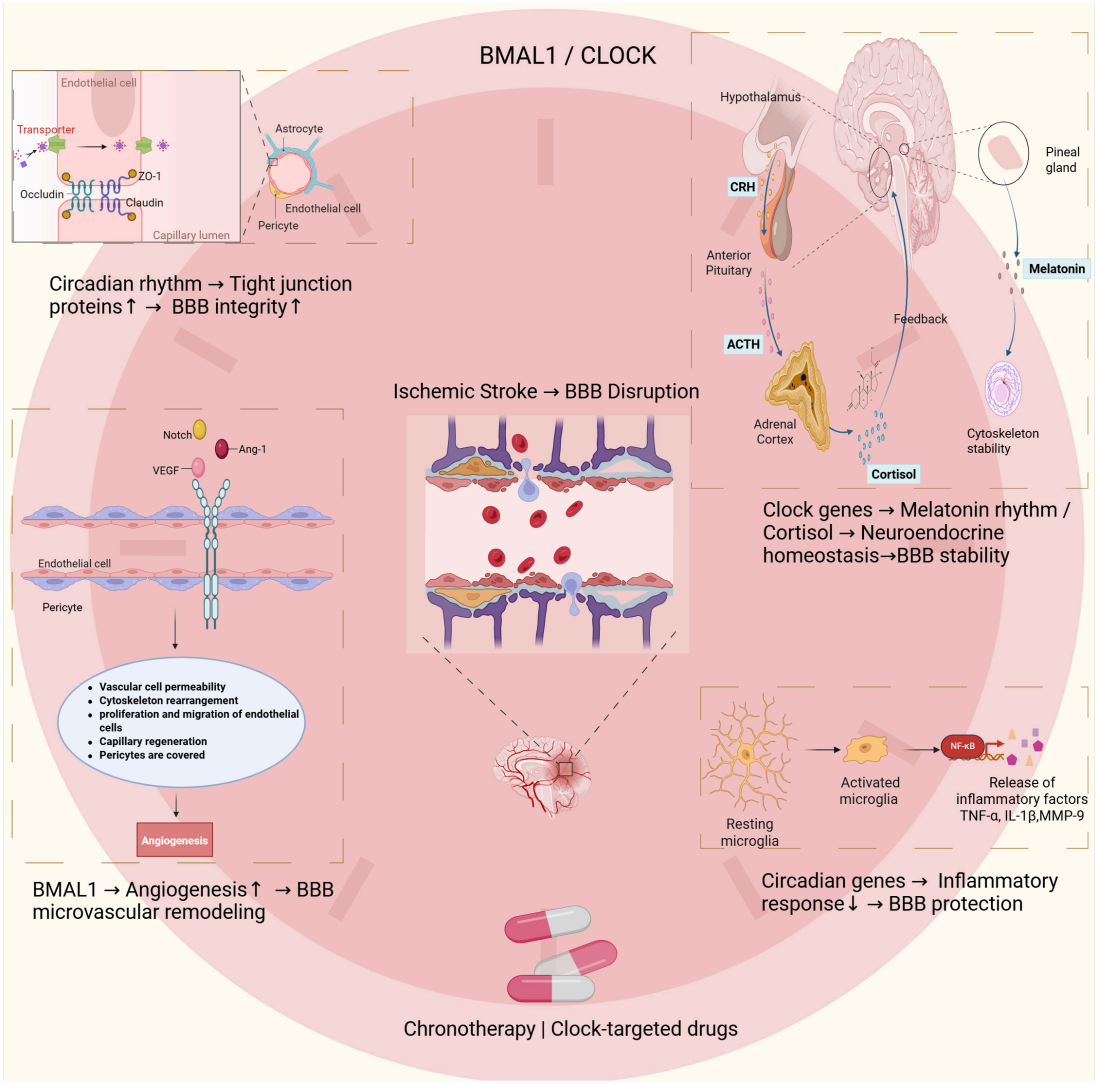
This schematic illustrates the interactions between circadian rhythms, blood–brain barrier (BBB) integrity, and ischemic stroke. The core circadian regulators BMAL1 and CLOCK play central roles in these processes. At the molecular level, circadian proteins maintain BBB integrity by regulating tight junction components such as occludin, claudin-5, and ZO-1. These effects are observed in endothelial cells, pericytes, and astrocytes. After ischemic injury, BMAL1 promotes angiogenesis by regulating vascular permeability, cytoskeletal remodeling, endothelial proliferation, and pericyte recruitment. These processes support microvascular remodeling and BBB restoration. Circadian genes also regulate neuroendocrine function, coordinating rhythmic melatonin and cortisol release. Clock genes modulate the hypothalamic–pituitary–adrenal (HPA) axis, mediated by CRH, ACTH, and cortisol, thereby influencing neuroendocrine balance and BBB function. Circadian regulation also limits neuroinflammation by restraining microglial activation and reducing the release of mediators such as TNF-α, IL-1β, and MMP-9. These mechanisms provide a rationale for developing chronotherapy and circadian-based pharmacological strategies to improve BBB outcomes after ischemic stroke.

The circadian clock acts as a key temporal regulator that preserves the structural and functional homeostasis of the BBB through integrated molecular networks. Here, we summarize the molecular mechanisms through which circadian rhythms regulate BBB repair after ischemic stroke (Figure 1). Key mechanisms involve transcriptional regulation of tight junction proteins, circadian control of angiogenesis, modulation of neuroimmune responses, and coordination of neuroendocrine signaling. Both preclinical and clinical studies show that circadian disruption aggravates BBB injury, characterized by reduced expression of tight junction proteins (ZO-1, occludin, claudin-5), heightened neuroinflammation (TNF-α, IL-1β), MMP-9/2–mediated extracellular matrix degradation, and HPA axis dysregulation. Emerging chronotherapeutic strategies—including melatonin receptor agonists, time-restricted feeding, and targeted temperature modulation—show translational potential for enhancing BBB repair after stroke. Circadian regulation influences all phases of drug disposition (ADME) and receptor availability, thereby shaping therapeutic efficacy across time. Recent studies highlight circadian-targeted approaches for BBB repair after stroke, including mesenchymal stem cell–derived exosomes (160) and herbal compounds (161, 162), which act in part by modulating clock gene expression. Chronopharmacological dosing that aligns with circadian fluctuations in BBB permeability may improve treatment outcomes. For instance, scheduling thrombolytic therapy to coincide with peak permeability could reduce hemorrhagic complications (163, 164), while nanocarrier-based systems show enhanced BBB penetration during specific circadian windows (165, 166). Additional non-pharmacological interventions, including photobiomodulation (167–169) and circadian-aligned feeding (170–172), can restore endogenous rhythmicity and offer new avenues for neurorehabilitation.

Despite recent progress, the mechanistic links between circadian rhythms and BBB dynamics remain incompletely understood, and current animal models are limited by interspecies physiological differences. Standardized methods for evaluating BBB injury and repair after ischemia are also lacking, further complicated by interindividual variability and uncontrolled confounders. Bridging these gaps will require interdisciplinary approaches that integrate three complementary axes: mechanistic dissection of circadian–BBB interactions, clinical validation of chronotherapeutic strategies, and the development of translational technologies. Key translational needs include stroke models with greater human relevance, practical tools for BBB monitoring, and therapeutic protocols optimized for circadian timing. Future work should emphasize high-resolution mapping of BBB rhythmicity, precision chronotherapy tailored to individual circadian signatures, and the evidence-based incorporation of traditional Chinese medicine (TCM) into circadian biology frameworks. In conclusion, advancing our understanding of circadian regulation of BBB repair will not only deepen insight into stroke pathogenesis but also enable the design of temporally targeted treatments. Integrating modern chronobiology with traditional chronomedicine offers a promising path toward more precise and effective interventions, with the potential to improve post-stroke recovery and long-term outcomes.
